# Photoluminescence associated with the site occupations of Ho^3+^ ions in BaTiO_3_

**DOI:** 10.1038/s41598-017-06521-4

**Published:** 2017-07-21

**Authors:** Da-Yong Lu, Dong-Xue Guan

**Affiliations:** 1 Research Center for Materials Science and Engineering, Jilin Institute of Chemical Technology, Jilin, 132022 P. R. China; 2 Key Laboratory of Special Functional Materials in Jilin Provincial Universities, Jilin, 132022 P. R. China; 3 College of Chemistry and Pharmaceutical Engineering, Jilin Institute of Chemical Technology, Jilin, 132022 P. R. China

## Abstract

A nominal (Ba_1−*x*_Ho_*x*_)Ti_1−*x*/4_O_3_ (*x* = 0.01) (BHTH) ceramic with a single-phase tetragonal structure was prepared at 1400 °C using the solid-state reaction method. The analysis on the defect chemistry revealed that the real formula of BHTH is (Ba_1−*x*_Ho_3*x*/4_)(Ti_1−*x*/4_Ho_*x*/4_)O_3_ with Ba vacancies via electron paramagnetic resonance (EPR). Photoluminescence (PL) was investigated on the basis of excitation with different wavelength lasers. The results indicated that under 488-nm excitation, PL and Raman scattering can occur simultaneously as two distinct optical processes for BHTH ceramic powders, and the strongest PL band at 564 nm was discovered and verified to originate from the ^5^G_6_/^5^F_1_ → ^5^I_7_ transition of Ho^3+^ ions on the Ti sites in the BaTiO_3_ lattice. Upon 532- and 638-nm excitations, three PL bands of ^5^F_4_/^5^S_2_ → ^5^I_8_, ^5^F_5_ → ^5^I_8_, and ^5^F_4_/^5^S_2_ → ^5^I_7_ transitions are attributed to the contributions from Ho^3+^ ions on the Ba sites. The common Raman spectrum of BaTiO_3_ can be observed without PL disturbance using 785-nm excitation wavelength. The PL effect may provide a probe for the site occupations of Ho^3+^ ions in widely-used BaTiO_3_ dielectric ceramics co-doped with Ho^3+^ and other dopants.

## Introduction

Ho^3+^, as a co-dopant with other dopants (Er^3+^, Tm^3+^, Yb^3+^, Dy^3+^) in compounds, is widely used in upconversion luminescence in the filed of luminescence^1–6^ and in multilayer ceramic capacitors (MLCC) in the filed of dielectrics^7, 8^. Barium titanate (BaTiO_3_) is a typical dielectric material. The dielectric properties and structural information of Ho-doped BaTiO_3_ were systematically investigated in terms of structural modification^9–15^. Ho^3+^ is known to be an amphoteric dopant in BaTiO_3_ polycrystalline ceramic and its solid solubility is dependent on the Ba/Ti ratio and sintering temperature (*T*_s_). In the Ti-rich case, Ho^3+^ was considered to to occupy the Ba site to induce Ti vacancies and the solid solubility limit was relatively small, only *x* = ~1.4% at *T*_s_ = 1400 °C; the formula was described as (Ba_1−*x*_Ho_*x*_)Ti_1−*x*/4_O_3_^10^. Higher *T*_s_ is generally required for the incorporation of Ho^3+^ into the Ti site because of a relatively larger ionic size of Ho^3+^ compared to Ti^4+^ (Table 1)^16^ and TiO_6_ octahedrons characteristic of the skeleton of the perovskite structure. In the Ba-rich case, the solubility limit was reported to be *x* = ~15% at *T*_s_ = 1550 °C and the formula was described as Ba(Ti_1−*x*_Ho_*x*_)O_3−*x*/2_ with O vacancies^10, 14, 15^. However, Ti vacancies and O ones had not been confirmed in Ho-doped BaTiO_3_ and one is hard to avoid questioning a single site-occupation mode due to the amphoteric nature of Ho^3+^.

**Table 1:** Ionic radii as a function of coordinate number (CN)^16.^

**Ion**	**CN**	**r (Å)**	**Ion**	**CN**	**r (Å)**
Ba^2+^	12	1.61	Ho^3+^	12	1.18
Ti^4+^	6	0.605	Ho^3+^	6	0.90

On the other hand, the structural information of BaTiO_3_ doped with most rare earths such as La^17–20^, Ce^21, 22^, Pr^23^, Nd^24^, Eu^25^, Tb^26^, Dy^27^, Er^28^, Yb^29, 30^, has been reported via laser Raman spectroscopy to date. However, no Raman information is known about Ho-doped BaTiO_3_. Because Ho usually acts as a well luminescent center in different host lattices^31–34^, the lack of Raman information about Ho-doped BaTiO_3_ is probably because the preference of strong photoluminescence (PL) caused by Ho^3+^ affects observation of Raman spectra, similar to Er^3+^ ^28^. As far as we know, only Battisha^35^ and Secu *et al*.^36^ reported the up-conversion luminescence (under 808-nm excitation) and PL (under 455-nm excitation) of Ho-doped BaTiO_3_. Some luminescence bands at 435 (^5^F_1_ → ^5^I_8_), 545 (^5^F_4_,^5^S_2_ → ^5^I_8_), 660 (^5^F_5_ → ^5^I_8_), and 760 nm (^5^F_4_,^5^S_2_ → ^5^I_7_) were observed. Unfortunately, XRD data show that their Ho-doped BaTiO_3_ samples prepared at *T*_s_ = 750 and 1350 °C are not single-phase, accompanied by a secondary phase such as Ho_2_Ti_2_O_7_^35, 36^. For this reason, the PL origin associated with the site occupations of Ho^3+^ ions in BaTiO_3_ is still an unresolved scientific problem. Moreover, a distinct exhibition between PL and Raman scattering needs to be also clarified.

In this work, a nominal (Ba_1−*x*_Ho_*x*_)Ti_1−*x*/4_O_3_ (*x* = 0.01) ceramic was prepared at 1400 °C using the solid-state reaction method. The defect chemistry is discussed. On the basis of excitation with different wavelength lasers (488‒785 nm), photoluminescence and Raman scattering can occur simultaneously as two distinct optical processes for BHTH ceramic powders. The reasonable laser wavelength is indicated for observation for the common Raman phonon modes of Ho-doped BaTiO_3_. A rare non-ground-state transition corresponding to ^5^G_6_/^5^F_1_ → ^5^I_7_ of Ho^3+^ was discovered and associated with the site occupations of Ho^3+^ ions in BaTiO_3_, which may provide a probe for the site occupations of Ho^3+^ ions in BaTiO_3_-based dielectric ceramics.

## Experimental

The initial materials were analytical-reagent chemicals of BaCO_3_, TiO_2_, and Ho_2_O_3_ (99.9%). Ho-doped BaTiO_3_ ceramics were prepared using the solid-state reaction method according to the nominal formula (Ba_1−*x*_Ho_*x*_)Ti_1−*x*/4_O_3_ (*x* = 0.01) named as BHTH. The weighed powders were dry-mixed and ground. Then, the powders were calcined at 1100 °C for 5 h to decarbonate. The calcined powders were mixed with a small amount of PVA aqueous solution and pressed at a pressure of 200 MPa into pellets (Φ12 mm). These pellets were sintered at *T*_s_ = 1400 °C for 12 h, and cooled at a cooling rate of −200 °C/h from 1400 °C to 700 °C and then furnace-cooled to room temperature. The pellets were densified into crack-free ceramics. In addition, a nominal (Ba_1−*x*_Ho_*x*_)Ti_1−*x*/4_O_3_ (*x* = 0.01) (BH1T) ceramic was prepared at *T*_s_ = 1300 °C for 12 h for analysis on site-occupation-related photoluminescence of Ho^3+^ ions in the BaTiO_3_ lattice.

Powder X-ray diffraction (XRD) data were recorded from 20° to 85° and in steps of 0.02° using a DX-2700 X-ray diffractometer (Dandong Haoyuan) at room temperature. Lattice parameters were calculated with MS Modeling (Accelry Inc.) using Cu Kα_1_ radiation (λ = 1.540562 Å). Microstructure was observed using an EVOMA 10 scanning electron microscope (Zeiss) operated at 15 keV. Photoluminescence (PL) and Raman spectra of the ceramic powders were recorded using a LabRAM XploRA Raman spectrometer (Horiba Jobin Yvon) with 532- (green) and 638-nm (red) lasers and an inVia Raman spectrometer (Renishaw) equipped with 488- (blue-purple) and 785-nm (red) lasers. The laser power level was adjusted to 0.1–10% (Filter) of the normal output of 25 mW because of a huge difference in spectral intensity. The accumulation time and resolution are 2 s and 2.7 cm^−1^, respectively. Electron paramagnetic resonance (EPR) measurements were performed at room temperature using an EMX Plus X-band spectrometer (Bruker) operating at 9.84 GHz. The gyromagnetic factor (*g*) was calculated by the relationship *hν*_0_ = *gβH*, where *h* is the Planck constant (*h* = 6.626 × 10^−34^ J·s), *ν*_0_ is the microwave frequency, *β* is the Bohr magnetron (*β* = 9.262 × 10^−24^ J/T), *H* is the magnetic field strength.

## Results and Discussion

### Crystal structure, site occupation and microstructure

**Figure 1:**
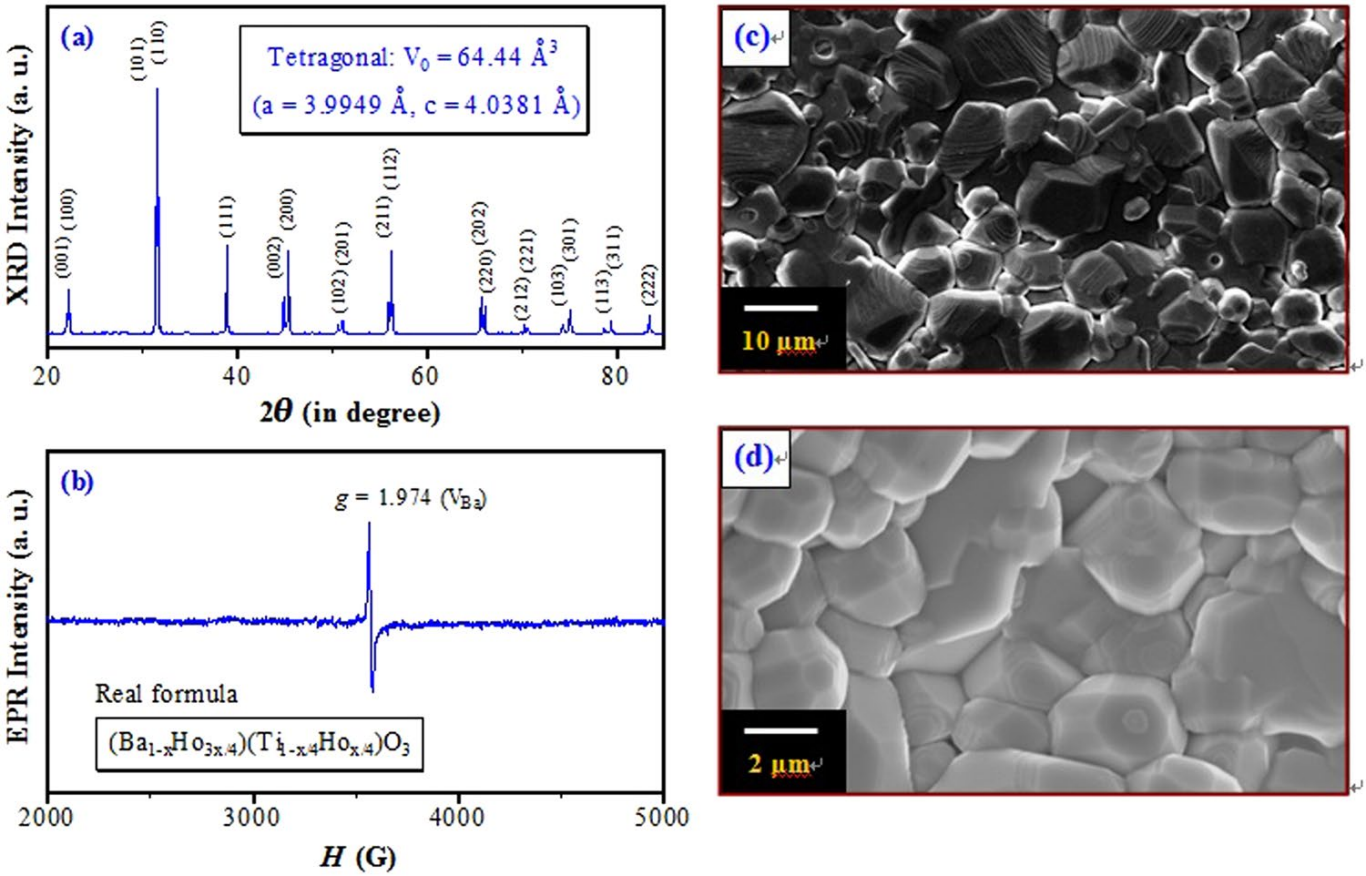
(**a**) XRD pattern, (**b**) EPR spectrum, SEM images on (**c**) the surface and (**d**) the interior for the nominal (Ba_1−*x*_Ho_*x*_)Ti_1−*x*/4_O_3_ (*x* = 0.01) ceramic (BHTH) sintered at a sintering temperature of *T*_s_ = 1400 °C. The real formula of BHTH is expressed by (Ba_1−*x*_Ho_3*x*/4_)(Ti_1−*x*/4_Ho_*x*/4_)O_3_ with Ba vacancies (V_Ba_).

Figure 1a shows the XRD pattern of (Ba_1−*x*_Ho_*x*_)Ti_1−*x*/4_O_3_ (*x* = 0.01) ceramic (BHTH) prepared at *T*_s_ = 1400 °C. BHTH has a single-phase tetragonal perovskite structure, indicating that Ho^3+^ ions are incorporated sufficiently into the perovskite lattice. This result is in accord with the solubility limit of *x* = 0.014 for (Ba_1−*x*_Ho_*x*_)Ti_1−*x*/4_O_3_ at *T*_s_ = 1400 °C reported by Makovec *et al*.^10^.

The lattice parameters (*a*, *c*) and unit cell volume (*V*_0_) is shown in Fig. 1a. It is inferred that the expansion in *V*_0_ caused by the occupations of the Ti^4+^ sites by Ho^3+^ ions should be greater than the contraction in *V*_0_ caused by the occupations of the Ba^2+^ sites by Ho^3+^ ions on the basis of the BO_6_ octahedrons skeleton characteristic of the perovskite lattice and ionic size comparisons between 12-CN Ba^2+^ and Ho^3+^ and between 6-CN Ti^4+^ and Ho^3+^ (Table 1). The *V*_0_ of BHTH (64.44 Å^3^) is slightly greater than that of the tetragonal BaTiO_3_ (*V*_0-BT_ = 64.41 Å^3^) (JCPDS Cards No. 6–526), implying that Ho^3+^ ions enter the Ti sites in part except for some Ho^3+^ ions on the Ba sites.

Figure 1b shows the EPR spectrum of BHTH. Ho^3+^ (4*f* ^10^) is a non-Kramers ion, which is EPR silent in theory. A very strong EPR signal at *g = *1.974 appears in BHTH and was observed in Dy- and Er-doped BaTiO_3_^27, 28^. This signal is assigned to ionized Ba-vacancy defects (V_Ba_)^27, 28, 37, 38^. A *g = *2.004 signal associated with Ti vacancies is absent for BHTH, revealing that Ti-vacancy defects cannot be formed in the nominal (Ba_1−*x*_Ho_*x*_)Ti_1−*x*/4_O_3_ (BHTH) with deliberately designed Ti vacancies; Ho^3+^ ions transfer from the Ba sites to the Ti ones and results in appearance of Ba vacancies in BHTH. This result is in good agreement with the previous XRD result.

Figure 1c and d shows the SEM images on the surface and the interior of BHTH. The surface of the ceramic sintered exhibits an inhomogeneous microstructure (Fig. 1c) with grains (2‒20 μm), but the interior in the ceramic shows a fine-grained feature (2‒6 μm) (Fig. 1d). This illustrates that the grain growth on the surface is faster than the interior.

### Defect chemistry

For the nominal (Ba_1−*x*_Ho_*x*_)Ti_1−*x*/4_O_3_ (*x* = 0.01) ceramic (BHTH) sintered at *T*_s_ = 1400 °C, the above XRD result gives evidence of a single-phase perovskite ceramic and the partial occupations of the Ti sites by Ho^3+^ ions (Fig. 1a); the EPR result reveals that Ti vacancies in BHTH are completely filled by Ho^3+^ and the transfer of Ho^3+^ ions from the Ba sites to the Ti ones gives rise to the presence of Ba vacancies (Fig. 1b). It is obvious that the formula cannot be described as (Ba_1−*x*_Ho_*x*_)Ti_1−*x*/4_O_3_ with Ti vacancies suggested by Makovec *et al*.^10^. Ho^3+^ is known to show an amphoteric behavior, substituting for both Ba and Ti sites in BaTiO_3_^10–12^. Thus, the defects in BHTH are Ba-site Ho^3+^ (${{\rm{Ho}}}_{{\rm{Ba}}}^{\bullet }$), Ba vacancies (${{\rm{V}}}_{{\rm{Ba}}}^{^{\prime\prime} }$), and Ti-site Ho^3+^ (${{\rm{Ho}}}_{{\rm{Ti}}}^{^{\prime} }$). The defect notation adopts that suggested by Kröger and Vink^39^. The lattice electroneutrality is collectively compensated by these three types of point defects. The detailed process of point defects formation is as follows:

Ho^3+^ ions enter both Ba and Ti sites simultaneously, forming the self-compensated ${{\rm{Ho}}}_{{\rm{Ba}}}^{\bullet }-{{\rm{Ho}}}_{{\rm{Ti}}}^{^{\prime} }$ defect complexes, similar to ${{\rm{Dy}}}_{{\rm{Ba}}}^{\bullet }-{{\rm{Dy}}}_{{\rm{Ti}}}^{^{\prime} }$ ^27^ and ${{\rm{Er}}}_{{\rm{Ba}}}^{\bullet }-{{\rm{Er}}}_{{\rm{Ti}}}^{^{\prime} }$ ^28^.

1 $${{\rm{Ho}}}_{{\rm{2}}}{{\rm{O}}}_{{\rm{3}}}\to {{\rm{Ho}}}_{{\rm{Ba}}}^{\bullet }+{{\rm{Ho}}}_{{\rm{Ti}}}^{^{\prime} }+{{\rm{3O}}}_{{\rm{O}}}$$

The partial occupations of the Ti sites by Ho^3+^ ions result in a Ti-rich case. Accordingly, Ba vacancies are induced by the extra Ba-site Ho^3+^ ions excluding ${{\rm{Ho}}}_{{\rm{Ba}}}^{\bullet }-{{\rm{Ho}}}_{{\rm{Ti}}}^{^{\prime} }$ to meet the requirement of the lattice electroneutrality.

2 $${{\rm{Ho}}}_{{\rm{2}}}{{\rm{O}}}_{{\rm{3}}}+3{{\rm{TiO}}}_{{\rm{2}}}\to 2{{\rm{Ho}}}_{{\rm{Ba}}}^{\bullet }+{{\rm{V}}}_{{\rm{Ba}}}^{^{\prime\prime} }+3{{\rm{Ti}}}_{{\rm{Ti}}}+{{\rm{9O}}}_{{\rm{O}}}$$

Thus, a very strong EPR signal at *g* = 1.974 is present in BHTH (Fig. 1b). The real formula of BHTH is determined to be (Ba_1−*x*_Ho_3*x*/4_)(Ti_1−*x*/4_Ho_*x*/4_)O_3_ with double-site substitutions and Ba-vacancy compensation, i.e., that at the doping level of *x* = 0.01, 0.75 at.% Ho^3+^ ions are substituted on the Ba sites and 0.25 at.% Ho^3+^ ions on the Ti sites. The quantitative information on the site occupations of Ho^3+^ ions in BHTH can be drawn from the EPR and XRD results. The defect chemistry of BHTH is the same as that of BaTiO_3_ doped with Dy^27^ and Er^28^. An interesting phenomenon is that Ho (AN = 67) is between Dy (66) and Er (68) in atomic number (AN).

### Photoluminescence and Raman spectra

**Figure 2:**
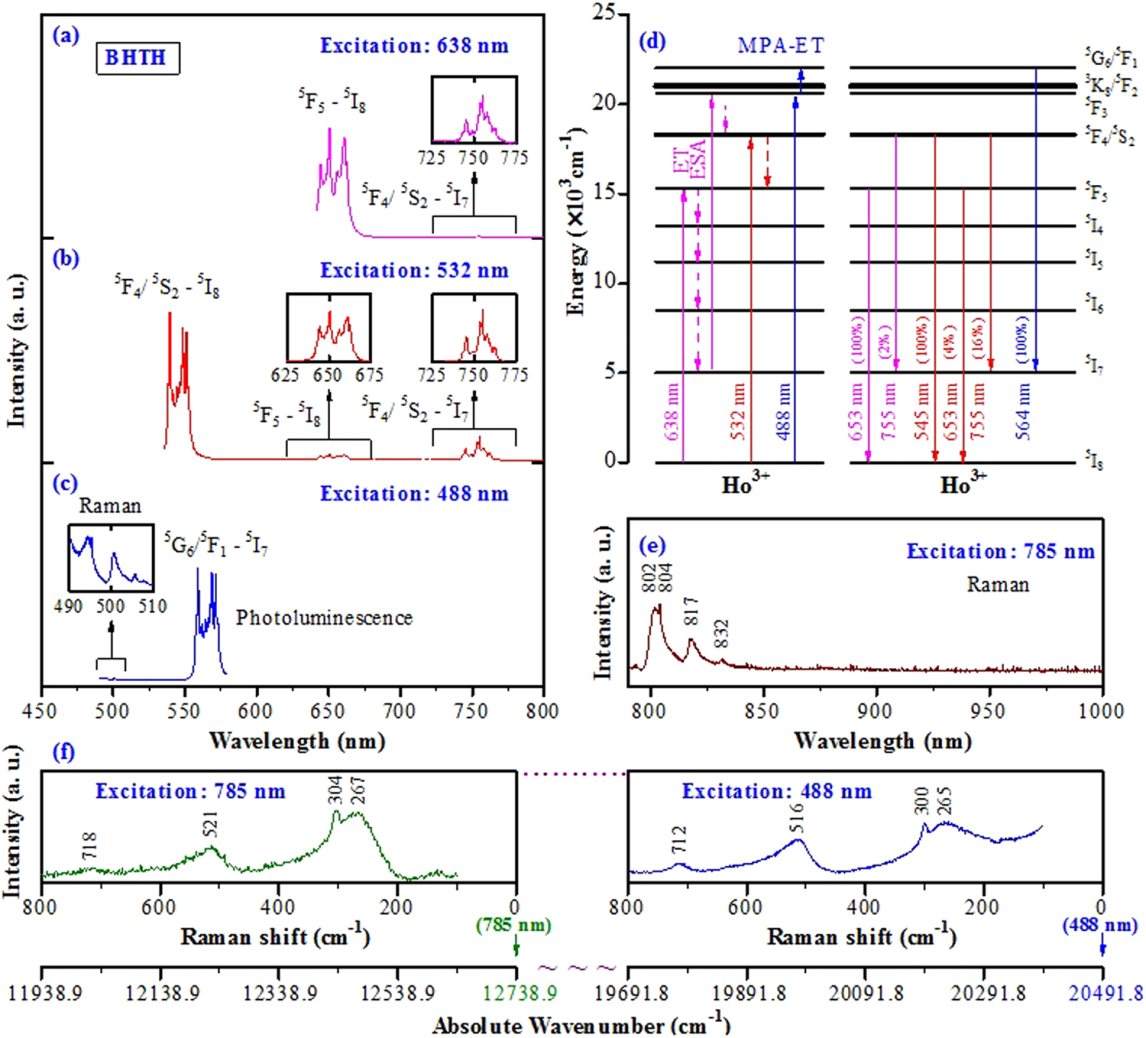
PL spectra of BHTH at room temperature, excitation with (**a**) 638-, (**b**) 532-, and (**c**) 488-nm laser lines. (**d**) Energy level diagram for Ho^3+^ ions in BHTH. MPA-ET represents multi-phonon assisted energy transfer. The values in bracket represent the percentage of the line intensity to the strongest emission line intensity. (**e**) Pure Raman spectrum is observed using 785-nm excitation wavelength. (**f**) Comparison of Raman spectra (stokes components) under 785- and 488-nm excitation wavelengths, shown in Raman shift and absolute wavenumber. The right figure shows the weak bands at around 500 nm in Fig. 2c inset.

Figure 2a‒c show the photoluminescence (PL) spectra of BHTH upon excitations with 638-, 532-, and 488-nm laser lines. Energy level diagram for Ho^3+^ ions in BHTH is shown in Fig. 2d. In the detectable range, these PL bands originate from the 4f-4f inner-shell emission of Ho^3+^ in BaTiO_3_. Upon 638-nm excitation, Ho^3+^ ions are excited through one-photon absorption from ^5^I_8_ to ^5^F_5_; the strongest emission band at 653 nm (Fig. 2a) is caused by the ^5^F_5_ → ^5^I_8_ transition. The near infrared (NIR) emission band (755 nm) associated with the ^5^F_4_/^5^S_2_ → ^5^I_7_ transition might originate from the following process: Ho^3+^ ions are excited through one-photon absorption from the ground state ^5^I_8_ to the excited state ^5^F_5_ that relaxes nonradiatively to the lying excited state ^5^I_7_ *via* continuous multi-phonon relaxation processes (^5^F_5_-^5^I_4_-^5^I_5_-^5^I_6_-^5^I_7_). Subsequently, Ho^3+^ ions were excited again through one-photon absorption (excited state absorption (ESA) process or energy transfer (ET) process) from ^5^I_7_ to the excited state ^5^F_3_ that relaxes nonradiatively to the emitting level ^5^F_4_/^5^S_2_. Finally, the ^5^F_4_/^5^S_2_ → ^5^I_7_ transition occurs (Fig. 2d). Each emission band shows a multiplet feature because of the Stark components of the ground state ^5^I_8_ and the excited states ^5^F_4_/^5^S_2_ and ^5^F_5_^5, 31, 32, 35^.

Upon 532-nm excitation, three PL bands with different intensities can be observed (Fig. 2b). The emission mechanism of Ho^3+^ in BHTH does not go through two-step ESA or ET process, but one-step one-photon absorption from ^5^I_8_ to ^5^F_4_/^5^S_2_ that relax nonradiatively to the emitting level ^5^F_5_, which is the reason that the intensity of the NIR (755 nm) emission band under 532-nm excitation is higher than that under 638-nm excitation. BHTH exhibits strong green (545 nm) with weak red (653 nm) and NIR (755 nm) emission bands associated with ^5^F_4_/^5^S_2_ → ^5^I_8_, ^5^F_5_ → ^5^I_8_ and ^5^F_4_/^5^S_2_ → ^5^I_7_ transitions, respectively, of Ho^3+^ ions^31, 32^. The multiplet feature of bands is also caused by Stark splitting of the energy levels. It can be seen from the insets in Fig. 2a and b that the corresponding emission bands upon excitation with different wavelengths exhibit the same spectral structure.

Pure Raman spectrum was observed in BHTH using 785-nm excitation wavelength, as shown in Fig. 2f. This spectrum of BHTH, which does not show any emission transition line and is similar to that reported for the tetragonal BaTiO_3_^28, 40–42^, shows four common bands, with peaks at 267 [A_1_ (TO_2_)], 304 [B_1_ + E(TO_3_ + LO_2_)], 521 [A_1_ (TO_3_)], and 718 cm^−1^ [A_1_ (LO_3_) + E (LO_4_)], corresponding to 802, 804, 817, and 832 nm in wavelength (Fig. 2e), respectively. No signal was present in the range of 850–1000 nm.

Upon 488-nm excitation, two bands were observed and the intensity of the main PL band at around 564 nm is 40 times that of the weak band at around 500 nm (Fig. 2c). The band at 564 nm, which has not been reported for Ho-doped BaTiO_3_^31, 32^, was observed for BHTH (Fig. 2c). This band is attributed to the ^5^G_6_/^5^F_1_ → ^5^I_7_ transition (Fig. 2d). The following analyses give its emission mechanism, and indicate that the band at 564 nm is associated with Ho^3+^ ions on the Ti sites and the weak band at 500 nm originates from Raman scattering of BHTH.

It is well known that when the excitation is changed to a different wavelength, the Raman bands shift the same amount correspondingly, while the PL lines stay on the same absolute wavenumber^28, 43^. Upon 638- and 532-nm excitations, it can be seen that both ^5^F_5_ → ^5^I_8_ and ^5^F_4_/^5^S_2_ → ^5^I_7_ transition bands at around 653 and 755 nm (Fig. 2a and b) stay on the same position, respectively, illustrating the PL nature of these two bands. Upon 488-nm (20491.8 cm^−1^) and 785-nm (12738.9 cm^−1^) excitations, Raman band is easy to be identified by comparison of band positions. Figure 2f shows their spectra, in which the right figure shows the weak band at 500 nm in Fig. 2c inset. When the excitation is changed from 488 nm to 785 nm, the shift of 7752.9 cm^−1^ between two Raman signals was observed for BHTH. Moreover, both spectra exhibit the nearly same spectral structure as the tetragonal BaTiO_3_ (Fig. 2f). Thus, the band at around 500 nm is confirmed to originate from Raman scattering in the case of excitation at 488 nm.

The ^5^G_6_/^5^F_1_ → ^5^I_7_ transition most likely originates from ground state absorption plus phonon-assisted transition (Fig. 2d). The ^5^F_3_ energy level can be populated from ^5^I_8_ via 488-nm laser energy absorption (one-photon). As the energy differences between ^5^F_3_ and ^3^K_8_/^5^F_2_ and between ^3^K_8_/^5^F_2_ and ^5^G_6_/^5^F_1_ is about 250 and 1050 cm^−1^, respectively, the multi-phonon assisted energy transfer (MPA-ET) corresponding to one A_1_ (TO_2_) phonon absorption (^5^F_3_ → ^3^K_8_/^5^F_2_) and then two A_1_ (TO_3_) phonons absorption (^3^K_8_/^5^F_2_ → ^5^G_6_/^5^F_1_) can occur. The absorbed phonons can be detected in light of the Raman spectrum in Fig. 2c inset or Fig. 2f. Normally, the three-phonon absorption is enough for the energy gap between ^5^F_3_ and ^5^G_6_/^5^F_1_. Finally, the emission band at 564 nm is due to the transition from ^5^G_6_/^5^F_1_ to the second ground state level ^5^I_7_.

On the basis of the above analyses, an important finding is that PL and Raman scattering under 488-nm excitation can occur simultaneously as two distinct optical processes for BHTH ceramic powders. One can also see that the PL signals from the ^5^F_4_/^5^S_2_ → ^5^I_8_ transition under 532-nm excitation are so intense that they overwhelm the traditional Raman spectra of BaTiO_3_ (Fig. 2b and Fig. 2c inset). The option for laser wavelength is an important factor in observation of Raman signals.

### Photoluminescent origin associated with site occupations of Ho^3+^ in BaTiO_3_

To investigate the PL origin associated with the site occupations of Ho^3+^ ions in the host BaTiO_3_ lattice, a BaTiO_3_ ceramic doped with Ho^3+^ on the Ba site is required to be prepared. In the Ti-rich case of Ba/Ti < 1, Ho^3+^ was considered to enter the Ba site for (Ba_1−*x*_Ho_*x*_)TiO_3_ sintered at *T*_s_ = 1320–1360°C in air^12–14^. For this reason, we prepared a nominal (Ba_1−*x*_Ho_*x*_)Ti_1−*x*/4_O_3_ (*x* = 0.01) ceramic (BH1T) sintered at *T*_s_ = 1300 °C. BHTH and BH1T have the same stoichiometric proportions in components.

**Figure 3:**
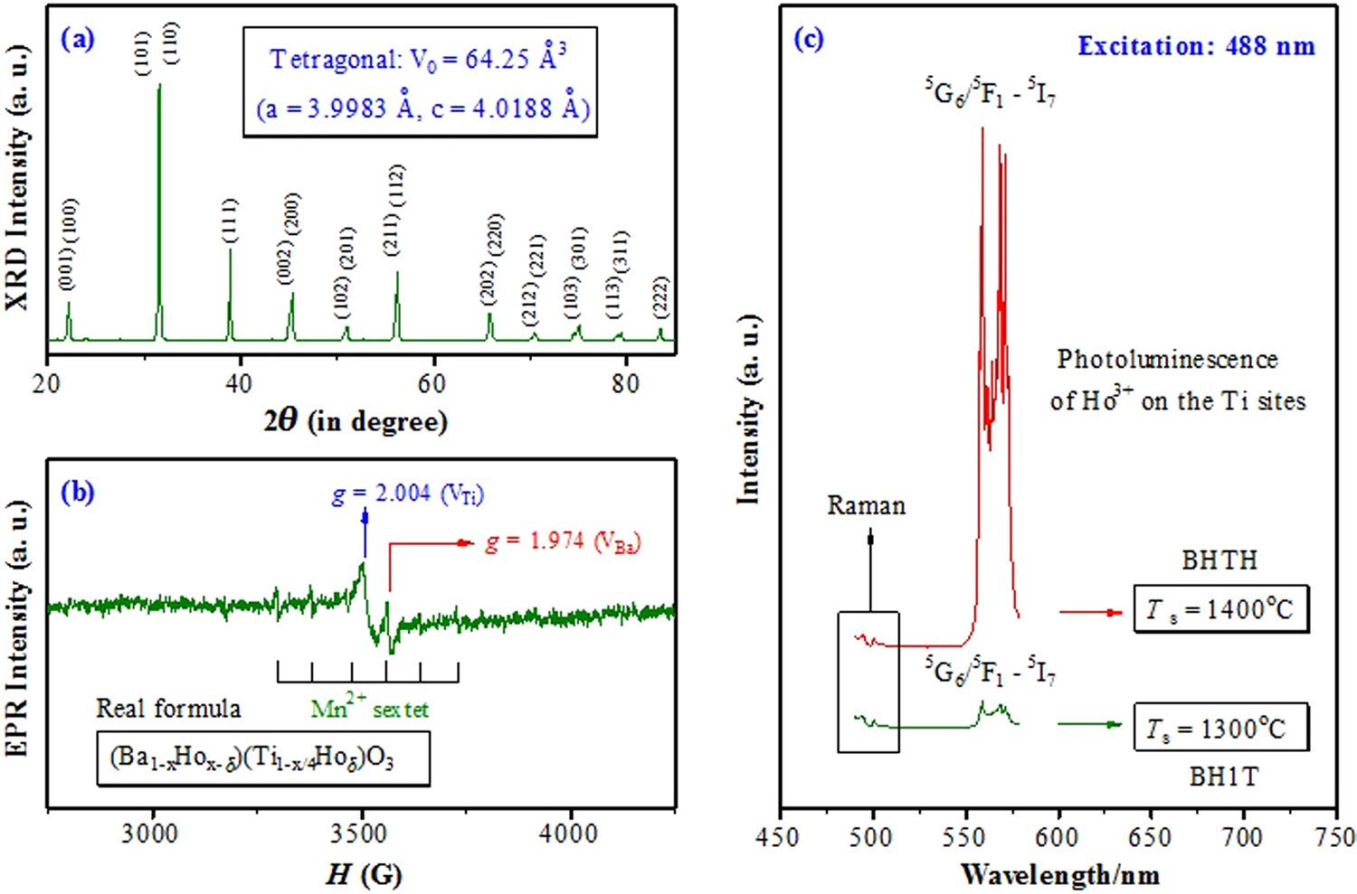
(**a**) XRD pattern, (**b**) EPR spectrum for the nominal (Ba_1−*x*_Ho_*x*_)Ti_1−*x*/4_O_3_ (*x* = 0.01) ceramic (BH1T) sintered at *T*_s_ = 1300 °C. The real formula of BH1T is approximately expressed by (Ba_1−*x*_Ho_*x*−*δ*_)(Ti_1−*x*/4_Ho_*δ*_)O_3_ (*δ* is a small quantity) with a small number of Ti-site Ho^3+^ ions. (**c**) The comparison in photoluminescence spectra between BHTH and BH1T clearly reveals that Ti-site Ho^3+^ ions are responsible for ^5^G_6_/^5^F_1_ → ^5^I_7_ transition of Ho^3+^.

The XRD pattern and EPR spectrum of BH1T as well as a comparison in PL spectra between BHTH and BH1T under 488-nm excitation are shown in Fig. 3. BH1T also exhibits a single-phase tetragonal perovskite structure (Fig. 3a) like BHTH, implying a complete incorporation of Ho^3+^ ions into the host BaTiO_3_ lattice. The *V*_0_ of BH1T (64.25 Å^3^) is less than those of BHTH (64.44 Å^3^) and the tetragonal BaTiO_3_ (64.41 Å^3^), suggesting that Ho^3+^ ions are dominantly substituted for the Ba sites.

The EPR monitoring for BH1T shows coexistence of both Ba vacancies and Ti ones, marked by two signals at *g = *1.974 and 2.004, respectively (Fig. 3b), implying that Ho^3+^ may transfer mutually between the Ba site and the Ti one during ceramic sintering and cooling. This behavior is similar to that of Lu^3+^-doped BaTiO_3_^44^. In this case more Ho^3+^ ions in BH1T can be present on the Ba^2+^ sites relative to BHTH, resulting in a stronger donor effect. The Mn^2+^ sextet signal is therefore caused by the reduction of Mn^4+^/Mn^3+^ to Mn^2+^ impurities in BH1T^45, 46^.

For Ho-doped BaTiO_3_ ceramics prepared by Battisha and Secu *et al*., Ho^3+^ is mainly substituted for the Ba sites because of a lower sintering temperature. They observed some luminescence bands at 435 (^5^F_1_ → ^5^I_8_), 545 (^5^F_4_/^5^S_2_ → ^5^I_8_), 660 (^5^F_5_ → ^5^I_8_), and 760 nm (^5^F_4_/^5^S_2_ → ^5^I_7_)^35, 36^. The solid solubility limit of Ho^3+^ ions on the Ba sites is less than *x* = ~1.4% at *T*_s_ = 1400 °C^10^. For our BHTH, 0.75 at.% Ho^3+^ ions are substituted on the Ba sites. Thus, the PL bands at 545, 653, and 755 nm (Fig. 2a and b) are attributed to Ho^3+^ ions on the Ba sites.

In a detectable wavelength range, one can see that both PL bands at around 564 nm are nearly the same in spectral structure and different in intensity for BHTH and BH1T, while there is no difference between both Raman bands in a lower-wavelength range at around 500 nm (Fig. 3c). It is obvious that the PL band at around 564 nm does not relate to Ho^3+^ ions on the Ba sites. The above XRD and EPR results reveal that Ho^3+^ ions in BH1T are dominantly substituted for the Ba sites, which is in good agreement with the site occupations of Ho^3+^ at a lower sintering temperature of *T*_s_ = 1320–1360°C in air^12–14^. Similar to the doping behavior of Dy^3+^ or Er^3+^ in BaTiO_3_^27, 28^, a small number of Ho^3+^ ions in BH1T inevitably enter the Ti sites. For our BHTH, 0.25 at.% Ho^3+^ ions are substituted on the Ti sites. The PL intensity is proportional to the concentration of Ho^3+^ ions. The PL intensity of the ^5^G_6_/^5^F_1_ → ^5^I_7_ transition of BH1T is one-twentieth of BHTH (Fig. 3c), which matches the concentration of Ho^3+^ ions on the Ti sites in BH1T and BHTH. Thus, Ho^3+^ ions on the Ti sites are responsible for the ^5^G_6_/^5^F_1_ → ^5^I_7_ transition at around 564 nm.

It is inferred from the PL intensity that 0.0125 at.% Ho^3+^ ions are substituted on the Ti sites in BH1T, though no quantitative information on the site occupations of Ho^3+^ ions on the Ti sites or Ba ones can be drawn directly from the EPR and XRD results. The real formula of BH1T may be approximately described as (Ba_1−*x*_Ho_*x*−*δ*_)(Ti_1-*x*/4_Ho_*δ*_)O_3_ (*δ* is a small quantity, *δ* = 0.0125%) with Ba vacancies and Ti ones. Hence, the change in local environment and crystal field at which Ho^3+^ lies plays a decisive role in PL.

### PL band at 564 nm as a probe for dielectrics in MLCC

Temperature-stable dielectric materials in MLCC often adopt the core-shell structured BaTiO_3_ ceramics, in which a fine grain consists of a tetragonal BaTiO_3_ core and a cubic surface layer consisting of Ho_2_O_3_ and other oxide, such as Ho/Mg^7, 47^, Ho/Mn^48^, Ho/Zr,^49, 50^ Ho/Dy^8^ etc. The incorporation of additives ions into BaTiO_3_ particles will reduce dielectric permittivity and dielectric-temperature stability. The sintering process plays an important role in controlling incorporation of Ho^3+^ into BaTiO_3_ particles. Although at a high sintering temperature (e.g. *T*_s_ = 1550 °C^10, 15^) Ho^3+^ ions are considered to substitute for the Ti sites, even at a lower *T*_s_ Ho^3+^ ions inevitably enter the Ti sites in part, as observed in Fig. 3c. It is more expensive and complicated to detect the amount of Ho^3+^ ions dissolved in the BaTiO_3_ lattice using an ion implantation technique with an accelerating voltage of 500 keV and secondary-ion mass spectrometry (SIMS)^11^. The PL of the ^5^G_6_/^5^F_1_ → ^5^I_7_ transition is sensitive to Ho^3+^ on the Ti sites in BaTiO_3_. Thus, the application of PL under 488-nm excitation may provide a probe for the occupations of Ho^3+^ ions on the Ti sites in BaTiO_3_ ceramics co-doped with Ho^3+^ and other dopants. Accordingly, the application of PL under 532- or 638-nm excitations may provide a probe for the occupations of Ho^3+^ on the Ba site.

## Conclusions

The nominal (Ba_1−*x*_Ho_*x*_)Ti_1−*x*/4_O_3_ (*x* = 0.01) (BHTH) ceramic was prepared at 1400 °C using the solid-state reaction method. BHTH exhibits a single-phase tetragonal perovskite structure. The study on the defect chemistry indicates that the defects in BHTH are Ba-site Ho^3+^ (${{\rm{Ho}}}_{{\rm{Ba}}}^{\bullet }$), Ba vacancies (${{\rm{V}}}_{{\rm{Ba}}}^{^{\prime\prime} }$), and Ti-site Ho^3+^ (${{\rm{Ho}}}_{{\rm{Ti}}}^{\text{'}}$). The real formula of BHTH is expressed by (Ba_1−*x*_Ho_3*x*/4_)(Ti_1−*x*/4_Ho_*x*/4_)O_3_. The change in local environment and crystal field at which Ho^3+^ lies plays a decisive role in photoluminescence (PL) of Ho^3+^ ions. Upon 532- and 638-nm excitations, three PL bands corresponding to ^5^F_4_/^5^S_2_ → ^5^I_8_, ^5^F_5_ → ^5^I_8_, and ^5^F_4_/^5^S_2_ → ^5^I_7_ transitions are attributed to the contributions from Ho^3+^ ions on the Ba sites in the BaTiO_3_ lattice. On the contrary, Ho^3+^ ions on the Ti sites are responsible for the ^5^G_6_/^5^F_1_ → ^5^I_7_ transition under 488-nm excitation, and moreover, PL and Raman scattering can occur simultaneously as two distinct optical processes. The common Raman spectrum of BaTiO_3_ can be observed without PL disturbance using 785-nm excitation wavelength. The PL signals under 532-nm excitation are intense enough to conceal the traditional Raman phonon modes of BaTiO_3_. The application of PL may provide a probe for the site occupations of Ho^3+^ in BaTiO_3_ dielectric ceramics co-doped with Ho^3+^ and other dopants.
